# Hoxa11 and Hoxd11 Regulate Chondrocyte Differentiation Upstream of Runx2 and Shox2 in Mice

**DOI:** 10.1371/journal.pone.0043553

**Published:** 2012-08-20

**Authors:** Stefanie Gross, Yvonne Krause, Manuela Wuelling, Andrea Vortkamp

**Affiliations:** Department of Developmental Biology, Center for Medical Biotechnology, University of Duisburg-Essen, Essen, Germany; Leibniz Institute for Age Research - Fritz Lipmann Institute (FLI), Germany

## Abstract

During limb development, posterior *Hox* genes of the *Hoxa*- and *Hoxd* cluster provide positional information along the limb axis. Here we report a new function for Hoxa11 and Hoxd11 in regulating the early steps of chondrocyte differentiation. We analyzed forelimbs of *Hoxa11^−/−^;d11^−/−^* and *Ulnaless* mice, which are characterized by specifically shortened zeugopods. By detailed morphological and molecular analyses, we show that loss of Hoxa11 and Hoxd11 in the ulna of both mutants leads to an arrest of chondrocyte differentiation at a step before the separation into round and columnar cells takes place. Furthermore, we demonstrate that Hoxa11 and Hoxd11 act upstream of *Runx2* and *Shox2*, two key regulators of chondrocyte differentiation. We hypothesize that Runx2 activates *Shox2* in early chondrocytes, which at later stages induces *Runx2* expression to regulate hypertrophic differentiation. These results give insight into mechanisms by which positional information might be translated into a specific bone pattern.

## Introduction

The long bones of vertebrates are formed by the process of endochondral ossification, during which a cartilage intermediate is established and subsequently replaced by bone [1]–[3]. Endochondral ossification is initiated by mesenchymal cells that condense and differentiate into chondrocytes, which form the cartilage anlagen of the future skeletal elements. The cartilage anlagen are surrounded by a layer of fibroblast-like cells, the perichondrium. In the center of the cartilage anlagen, the cells start to successively differentiate into several types of chondrocytes, each showing a characteristic cell shape. Initially, the proliferating chondrocytes separate into two cell types: round, low-proliferating chondrocytes at the distal end of the skeletal element and columnar, high-proliferating chondrocytes in the center [4], [5]. The columnar chondrocytes are flattened and arranged in columns along the longitudinal axis. Once the chondrocytes stop proliferating, they differentiate into hypertrophic chondrocytes, which finally undergo apoptosis. During hypertrophic differentiation the chondrocytes increase their volume and produce a mineralized extracellular matrix. In parallel with hypertrophic differentiation, cells of the perichondrium differentiate into osteoblasts forming the periosteum. From the periosteum, blood vessels invade the hypertrophic region providing osteoblasts and osteoclasts to replace the cartilage by bone [1]–[3].

*Hox* genes belong to the highly conserved family of homeobox transcription factors that play a critical role in defining regional identity along the primary body and limb axis during embryonic development [6], [7]. In mice, 39 *Hox* genes are organized in 4 clusters (*Hoxa-Hoxd*), each of which is located on a different chromosome. Based on sequence similarities and the relative position in the cluster, *Hox* genes are divided into 13 paralogous groups [8], [9]. The formation of limbs is mainly regulated by posterior genes of the *Hoxa*- and *Hoxd*-cluster (*Hoxa9-a13*, *Hoxd9-d13*).

The expression of *Hox* genes is tightly regulated in time and space. Along the anterior-posterior body and limb axis the order of *Hox* gene expression reflects their arrangement on the chromosome with 3′ genes being expressed earlier and more anteriorly than 5′ genes [10], [11]. During limb development, the expression of *Hoxd* genes is under the control of two enhancers that are located on either side of the *Hoxd* cluster [12]–[16]. In a first phase of *Hoxd* gene expression, the colinear activation is regulated by the “Early Limb Control Region” (ELCR), which is located at the 3′end of the cluster. During this phase, the *Hoxd* genes are successively expressed, starting with *Hoxd1* being expressed throughout the early limb bud and ending with the expression of posterior *Hox* genes progressively restricted to the posterior region of the limb bud. In a second phase of *Hoxd* gene expression, only the posterior *Hoxd* genes (*Hoxd9-d13*) are expressed in reverse colinearity. This phase is regulated by the “Global Control Region” (GCR) that is located at the 5′ side of the *Hoxd* cluster [13], [15], [16]. This nested expression pattern of *Hoxd* genes in combination with genes of the *Hoxa* cluster generates a “*Hox* code” that specifies the identity of the limb skeletal elements [17].

Mutations of posterior *Hoxa* and *Hoxd* genes cause distinct limb deformities. Loss of single *Hox* genes leads to mild alterations of individual skeletal elements, whereas the deletion of additional paralogous *Hox* genes enhances the severity of the phenotype. For example, loss of Hoxa11 or Hoxd11 in mice leads to mild alterations of ulna and radius [18], [19], whereas compound *Hoxa11^−/−^;d11^−/−^* mice display a severe shortening of ulna and radius. Other skeletal elements develop more or less normally [20], [21]. A similar forelimb phenotype has been observed in the dominant mouse mutant *Ulnaless*, which carries a radiation induced inversion of the complete *Hoxd* cluster [13], [22]–[26]. Due to this inversion, the GCR is separated from the *Hoxd* cluster [13] leading to an altered temporal and spatial activation of posterior *Hoxd* genes. As a result, *Hoxd12* and *Hoxd13* are ectopically expressed in the zeugopod region, whereas the expression of *Hoxa11* and *Hoxd11* is significantly reduced [24], [25]. Based on the similarities of the forelimb phenotype and the loss of *Hoxa11* and *Hoxd11* expression, *Ulnaless* mice have been hypothesized to mimic the *Hoxa11^−/−^;d11^−/−^* mutation.

In this study, we investigated the molecular mechanisms acting downstream of *Hox* genes by analyzing chondrocyte differentiation in ulna and radius of *Hoxa11^−/−^;d11^−/−^* and *Ulnaless* mutant mice. Since the *Ulnaless* mutation is dominant (50% mutant embryos), this mouse strain facilitates the analysis of the phenotype compared to the double homozygous *Hoxa11^−/−^;d11^−/−^* embryos (6.25% mutant embryos).

By detailed morphological and molecular analyses, we show that the ulna is similarly affected in both mutants. Although chondrogenesis in the cartilage anlagen is initiated similar as in wild type mice at E12.5, the differentiation of chondrocytes is inhibited at an early step of the differentiation program at least until E16.5, when wild type limbs clearly have undergone hypertrophic differentiation. Molecular analyses indicate that the differentiation into round and columnar chondrocytes is disturbed. Furthermore, *Shox2* and *Runx2* expression cannot be detected in the mutant ulna, indicating that they act downstream of posterior *Hox* genes during chondrocyte differentiation.

## Results and Discussion

### The Ulna of *Ulnaless* Mice Serves as a Model for the *Hoxa11^−/−^;d11^−/−^* Mutation

**Figure 1:**
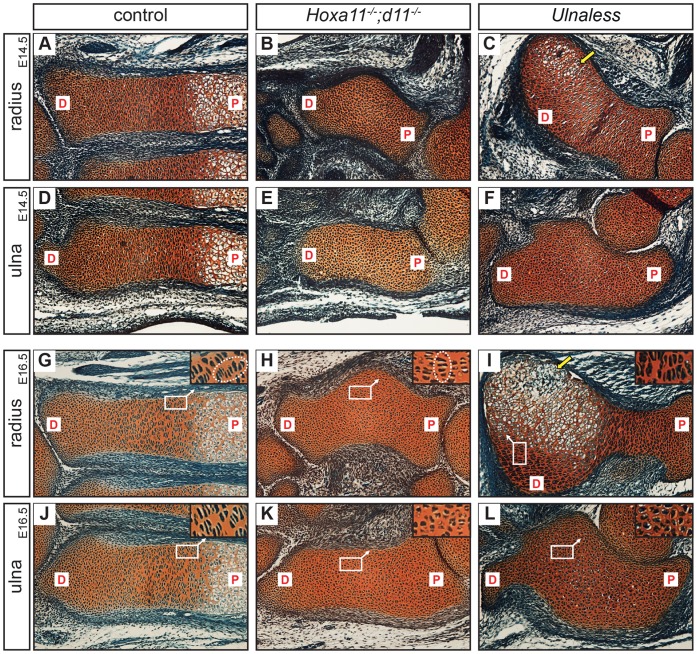
Lack of hypertrophic differentiation in the ulna of *Hoxa11^−/−^;d11^−/−^* and *Ulnaless* forelimbs. Safranin-Weigert staining of E14.5 (A–F) and E16.5 (G–L) control (A, D, G, J), *Hoxa11^−/−^;d11^−/−^* (B, E, H, K) and *Ulnaless* (C, F, I, L) forelimb sections reveals disturbed chondrocyte differentiation. Hypertrophic chondrocytes are absent in ulna (E, K) and radius (B, H) of *Hoxa11^−/−^;d11^−/−^* mice and in the ulna (F, L) of *Ulnaless* mice. Only in the curve of the radius of E14.5 *Ulnaless* mice hypertrophic chondrocytes are detectable (C, arrow). At E16.5, mineralized matrix is produced in the radius of *Ulnaless* mice (I, arrow), while in the radius of *Hoxa11^−/−^;d11^−/−^* mice columnar chondrocytes are formed (H). These cells are not organized in columns along the longitudinal axis as in the control, but in anterior-posterior direction (G, H, see encircled columns in higher magnification of boxed regions). 160x magnification; P = proximal, D = Distal.

Previously, it has been reported that *Hoxa11^−/−^;d11^−/−^* mice display a defect in hypertrophic chondrocyte differentiation in ulna and radius [20]. To gain insight into the molecular mechanisms acting downstream of *Hox* genes in chondrocytes, we investigated the process of chondrocyte differentiation in *Hoxa11^−/−^;d11^−/−^* mice at different developmental stages in detail and compared it to that of *Ulnaless* mutants. To confirm the similarity of the *Hoxa11^−/−^;d11^−/−^* and *Ulnaless* forelimb phenotypes, we analyzed forelimb sections of both mutants on a morphological level after Safranin-Weigert staining. At E14.5 and E16.5, ulna and radius of both mutants are significantly shortened compared to wild type limbs (Fig. 1). In addition, the radius is bent towards the anterior side (Fig. 1B, C, H, I). At both stages, the wild type cartilage elements of both zeugopod bones show well organized zones of chondrocytes: round chondrocytes located at the distal end of the cartilage element are followed by columnar and hypertrophic chondrocytes towards the center (Fig. 1A, D, G, J). In contrast, in the ulna of *Hoxa11^−/−^;d11^−/−^* mice no columnar or hypertrophic chondrocytes are detectable at E14.5 or E16.5 (Fig. 1E, K). In the radius hypertrophic differentiation is delayed at both stages, but a few columnar chondrocytes can be detected in the anterior curve at E16.5 (Fig. 1H). Interestingly, while columns in the radius of the control limb are aligned along the longitudinal axis, they are arranged in anterior-posterior direction in the *Hoxa11^−/−^;d11^−/−^* mutant radius (Fig. 1G, H, higher magnification of boxed regions). In the ulna of *Ulnaless* mutants hypertrophic differentiation is similarly blocked as in the ulna of *Hoxa11^−/−^;d11^−/−^* mutants (Fig. 1E, F, K, L). However, chondrocyte differentiation in the radius of *Ulnaless* mutants is less severely affected. Few cells with hypertrophic appearance can be detected at E14.5 and a distinct hypertrophic region is found at E16.5 (Fig. 1C arrow, I).

**Figure 2:**
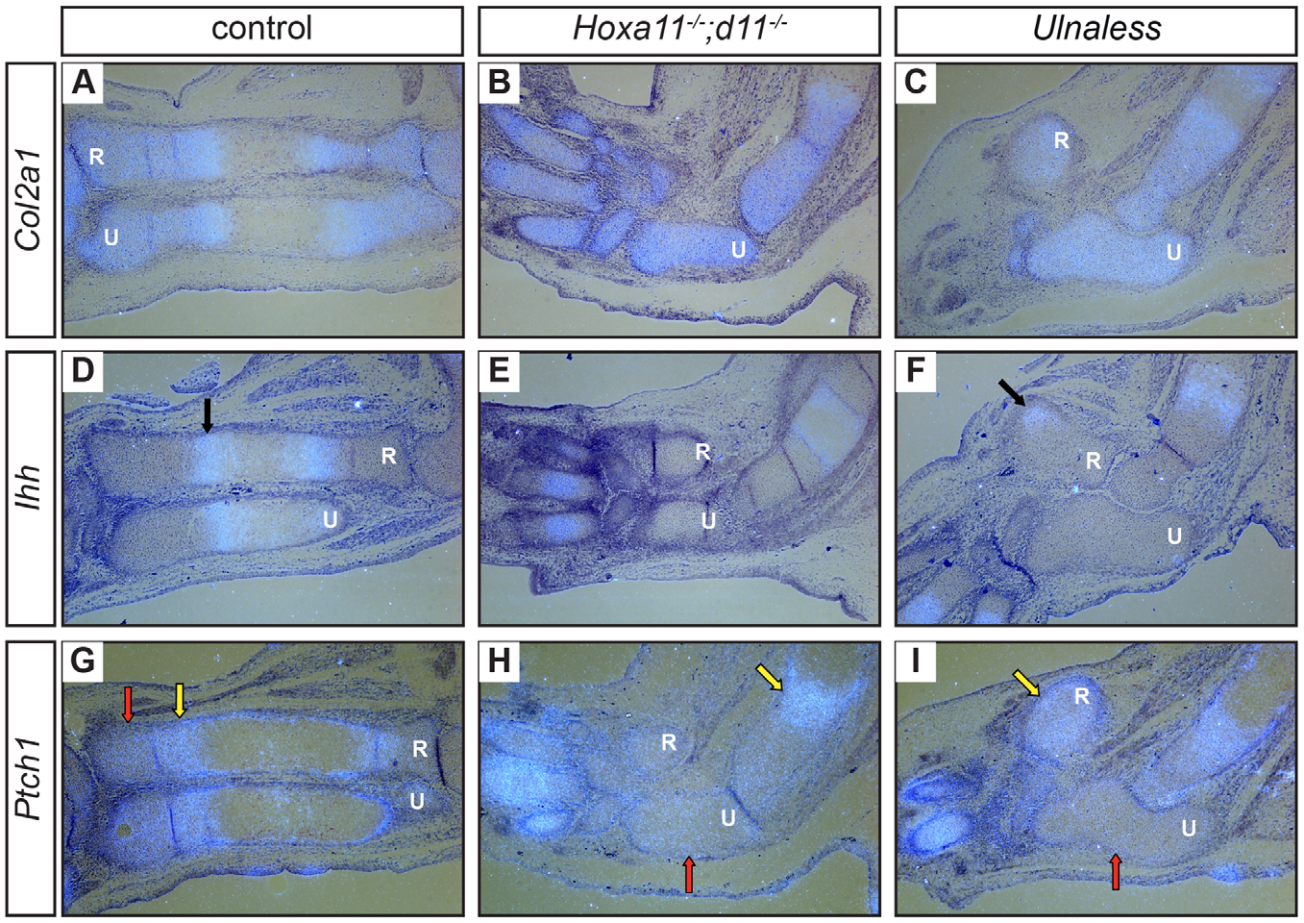
Chondrocyte differentiation is blocked prior to the separation into columnar and round chondrocytes. Sections of E14.5 forelimbs of control (A, D, G), *Hoxa11^−/−^;d11^−/−^* (B, E, H) and *Ulnaless* embryos (C, F, I) were hybridized with antisense riboprobes for *Col2a1* (A–C), *Ihh* (D–F) and *Ptch1* (G–I). At E14.5, *Col2a1* is expressed in all chondrocytes of ulna and radius of *Hoxa11^−/−^;d11^−/−^* and *Ulnaless* mutants similar as in control limbs (A–C). In contrast, *Ihh*, which is expressed in early hypertrophic chondrocytes in E14.5 control limbs (D, arrow), is not expressed in the ulna of both mutants (E, F). *Ptch1* is strongly expressed in wild type chondrocytes adjacent to the *Ihh* expression domain (G, yellow arrow), but only basal expression levels are detected in chondrocytes of the ulna of *Hoxa11^−/−^;d11^−/−^* and *Ulnaless* mice (G–I, red arrows). 80x magnification; R = radius, U = ulna.

To further characterize the chondrocyte differentiation defect on a molecular level, we analyzed the expression of genes demarcating distinct chondrocyte subpopulations by *in situ* hybridization. The extracellular matrix protein *Type II collagen* (*Col2a1*) and the transcription factor *SRY-box containing gene 9* (*Sox9*) are markers for cartilage condensations and proliferating chondrocytes [27]–[29] (Fig. 2A and data not shown). Although in *Hoxa11^−/−^;d11^−/−^* mice the condensations of ulna and radius are smaller than in wild type mice [20] (Fig. 1), *Col2a1* and *Sox9* are expressed in all chondrocytes at E14.5 (Fig. 2B and data not shown). A similar expression pattern of *Col2a1* and *Sox9* was detected in E14.5 *Ulnaless* mice (Fig. 2C and data not shown) indicating that cells condense and differentiate into chondrocytes. *Indian hedgehog* (*Ihh*), a key regulator of endochondral ossification, is expressed in early hypertrophic chondrocytes of E14.5 and E16.5 wild type limbs [30], [31] (Fig. 2D, arrow and data not shown). Corresponding to the morphological analysis (Fig. 1), no hypertrophic, *Ihh*-expressing cells are found in the ulna of *Hoxa11^−/−^;d11^−/−^* or *Ulnaless* mutants at E14.5 and E16.5 (Fig. 2E, F and data not shown) [20]. However, cells in the radius of *Ulnaless* mice that appear hypertrophic by morphology (Fig. 1C, arrow) express *Ihh* at E14.5 (Fig. 2F, arrow).

To exclude an early delay in chondrogenesis as the cause for the inhibition of hypertrophic differentiation we analyzed *Col2a1* expression in E12.5 *Ulnaless* mice. Similar to data published for E12.5 *Hoxa11^−/−^;d11^−/−^* mice [20], we detected no significant difference in *Col2a1* expression in the zeugopod of E12.5 *Ulnaless* mice ( Fig. S1) indicating that chondrocyte differentiation is properly initiated.

In summary, although the initial condensation of chondrocytes is not severely affected, we detected a severe inhibition of further chondrocyte differentiation in the zeugopod of *Hoxa11^−/−^;d11^−/−^* mutants that is maintained at least until E16.5. A comparable delay in chondrocyte differentiation was detected in the zeugopod of *Ulnaless* mice. Interestingly, chondrocyte differentiation seems to be more severely delayed in the ulna of both mutants, whereas the radius is less affected. To understand the role of posterior *Hox* genes in regulating chondrocyte differentiation, we thus concentrated our analysis on the ulna of both mutants.

### Chondrocytes in the Ulna of E13.5 *Ulnaless* Mice are not Competent to Undergo Hypertrophic Differentiation

**Figure 3:**
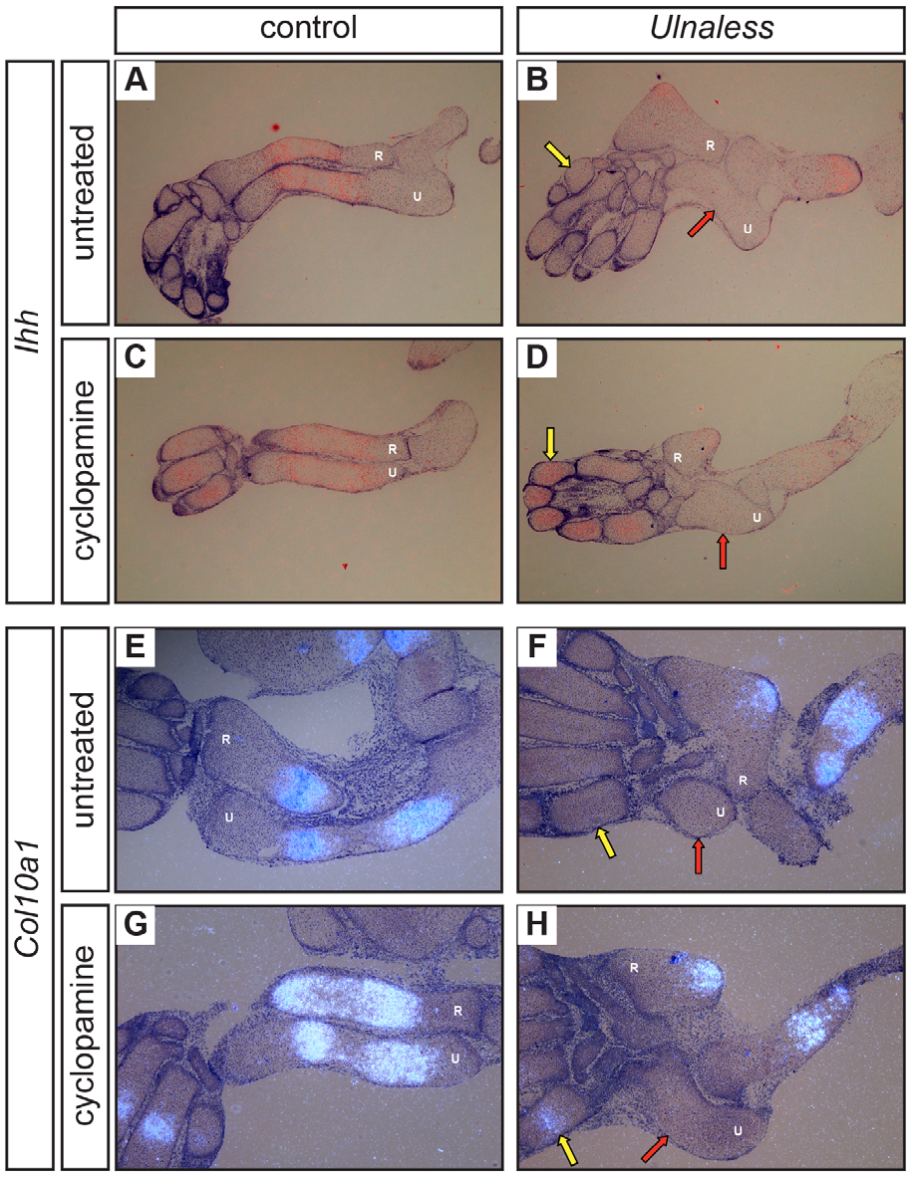
Chondrocytes in the *Ulnaless* ulna do not undergo hypertrophy upon blocking Ihh signaling. Sections of E13.5 control (A, C, E, G) and *Ulnaless* forelimbs (B, D, F, H) that had been cultured for 4 days in control medium (A, B, E, F) or in medium supplemented with cyclopamine (C, D, G, H), were hybridized with the antisense probes for *Ihh* (A–D) and *Col10a1* (E–H). Premature activation of *Ihh* as well as *Col10a1* expression can be detected in the digits of cyclopamine treated control (C, G) and *Ulnaless* (D, H, yellow arrows) forelimbs. In contrast, in the ulna of *Ulnaless* mice *Ihh* and *Col10a1* expression was not induced by cyclopamine (D, H, red arrows). 50x magnification (A–D), 80x magnification (E–H); R = radius, U = ulna.

It has previously been described that *Ihh*-deficient mice undergo accelerated hypertrophic differentiation [31]. Here we show that despite the lack of *Ihh* expression in the ulna of the *Hox* mutants at E14.5 and E16.5, no sign of hypertrophic differentiation can be detected (Fig. 1E, F, K, L; Fig. 2E, F and data not shown). To exclude that the level of *Ihh* expression is below the sensitivity of the *in situ* hybridization, we analyzed the expression of the Ihh receptor *Patched homolog 1* (*Ptch1*), which is highly expressed in cells receiving a Hedgehog signal. In E14.5 wild type skeletal elements, *Ptch1* is expressed in all proliferating chondrocytes with highest expression level adjacent to the *Ihh* expression domain [31] (Fig. 2G, yellow arrow). However, in the ulna of both *Hox* mutants, *Ptch1* expression is only detected at basal expression levels (Fig. 2H, I, red arrows). To further exclude residual levels of Ihh activity, we treated E13.5 forelimb explants of the dominant *Ulnaless* mutant with cyclopamine, which inhibits Ihh signaling thereby accelerating hypertrophic differentiation [32], [33]. In cyclopamine treated wild type cultures, hypertrophic differentiation was accelerated, as indicated by the increased expression domain of the hypertrophic marker *Ihh* and the shortened zone of proliferating chondrocytes in ulna, radius and in the digits (Fig. 3A, C). In contrast, in cyclopamine treated explant cultures of *Ulnaless* mice, we did not observe any *Ihh*-expressing hypertrophic chondrocytes in the ulna (Fig. 3B, D, red arrows), although *Ihh* expression was induced in the digits (Fig. 3B, D, yellow arrows). To confirm that hypertrophic differentiation is blocked in the mutants, we analyzed the expression of *Type X collagen* (*Col10a1*), another marker specifically expressed in hypertrophic cells [34]. Similar to *Ihh*, we did not detect any *Col10a1*-expressing chondrocytes in the ulna of cyclopamine treated *Ulnaless* limbs (Fig. 3F, H, red arrows), but observed accelerated hypertrophic differentiation in the digits (Fig. 3F, H, yellow arrows).

We can thus conclude that chondrocytes in the ulna of E13.5 *Ulnaless* mice are not competent to initiate hypertrophic differentiation upon lack of Ihh signaling.

Furthermore, it is interesting to note that the differentiation defect is maintained in the serum free organ cultures, in which the surrounding tissue is rapidly lost. We thus hypothesize that information inhibiting chondrocyte differentiation in the ulna is contained in the skeletal element itself, either in the perichondrium or in the chondrocytes.

### Hoxa11 and Hoxd11 Control Early Steps of Chondrocyte Differentiation

**Figure 4:**
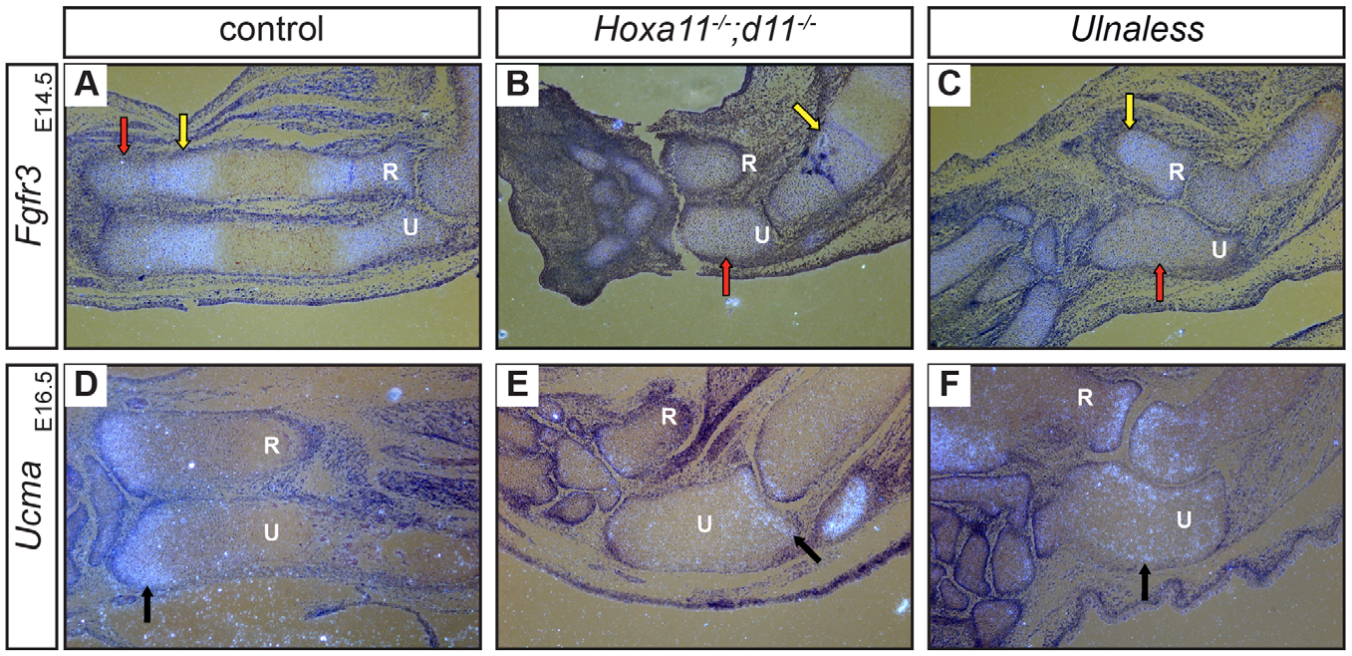
Hoxa11 and Hoxd11 control early steps of chondrocyte differentiation. Sections of E14.5 (A–C) and E16.5 (D–F) forelimbs of control (A, D), *Hoxa11^−/−^;d11^−/−^* (B, E) and *Ulnaless* embryos (C, F) were hybridized with antisense riboprobes for *Fgfr3* (A–C) and *Ucma* (D–F). High *Fgfr3* expression (A–C, yellow arrows), which demarcates columnar chondrocytes in control limbs (A), is significantly reduced in the ulna of the *Hox* mutants (B, C). The observed *Fgfr3* expression level is comparable with the expression in round chondrocytes of the control mice (A–C, red arrows). *Ucma*, a marker for round cells (D, arrow), is not expressed in the majority of chondrocytes indicating that the differentiation is blocked before round and columnar chondrocytes are formed (E, F, arrows). 80x magnification; R = radius, U = ulna.

Since loss of Hoxa11 and Hoxd11 impairs chondrocyte differentiation, we wished to identify the specific step of the differentiation program affected in the *Hox* mutants. As described above, proliferating chondrocytes can be subdivided into round and columnar cells. To define the subpopulations of proliferating chondrocytes in the mutants, we analyzed the expression of *Fibroblast growth factor receptor 3* (*Fgfr3)*. In E14.5 wild type limbs, *Fgfr3* is expressed at high levels in columnar chondrocytes (Fig. 4A, yellow arrow) and at lower levels in round cells (Fig. 4A, red arrow) [35]. In the ulna of both *Hox* mutants, we observed only low levels of *Fgfr3* expression (Fig. 4B, C, red arrows) indicating that the separation into round and columnar chondrocytes had not taken place. This supports the morphological observation that no columnar chondrocytes are formed. To further test if the chondrocytes represent the round cell type, we analyzed the expression of *Upper zone of growth plate and matrix associated protein* (*Ucma*), which is strongly expressed in these cells at E16.5 in wild type skeletal elements [36] (Fig. 4D, arrow). Surprisingly, in both mutants the majority of chondrocytes in the ulna do not express *Ucma*. Instead, *Ucma* expression is restricted to the joint region in the ulna of *Hoxa11^−/−^;d11^−/−^* mice (Fig. 4E, arrow) and displays a diffuse pattern in single chondrocytes in *Ulnaless* mice (Fig. 4F, arrow).

Together these results demonstrate that the chondrocyte differentiation process in both *Hox* mutants is disturbed before the differentiation into round and columnar chondrocytes takes place.

### *Shox2* and *Runx2* Act Downstream of Hoxa11 and Hoxd11

**Figure 5:**
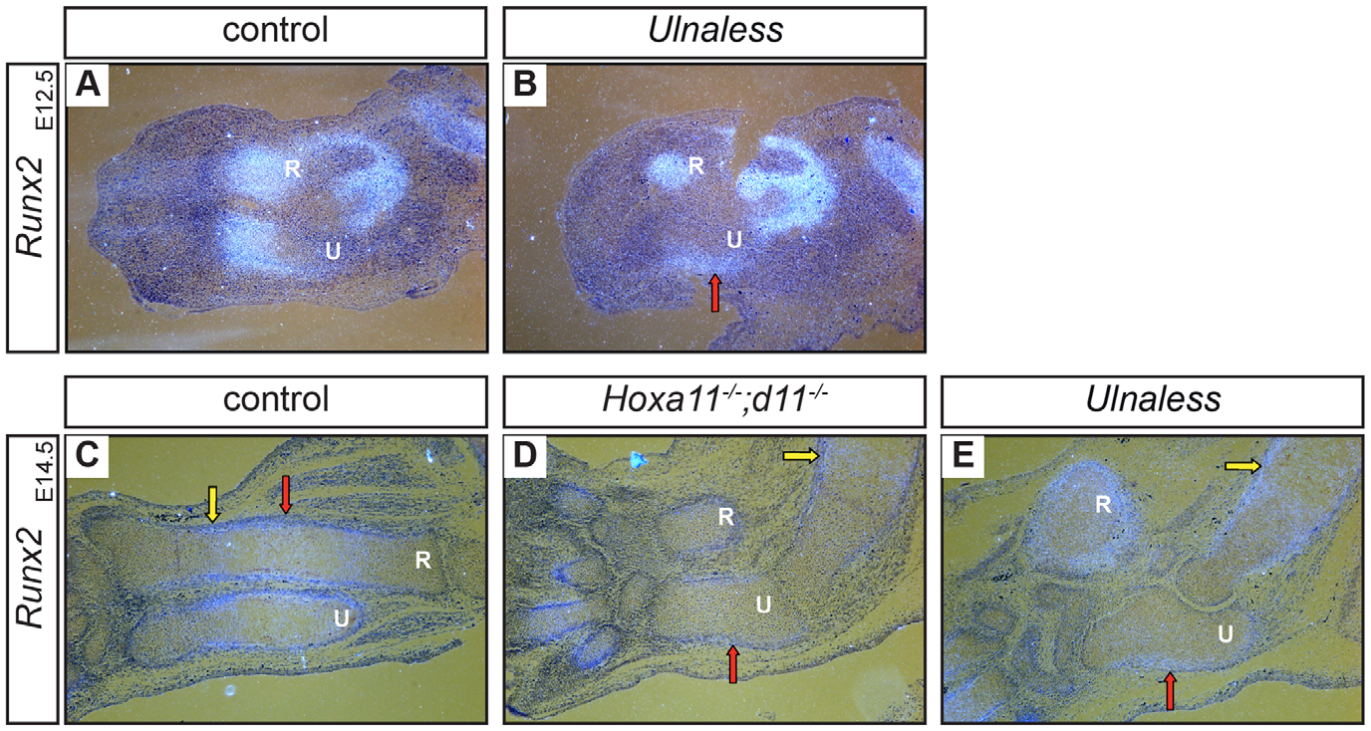
Runx2 acts downstream of Hoxa11 and Hoxd11. Sections of E12.5 (A, B) and E14.5 (C–E) control (A, C), *Hoxa11^−/−^;d11^−/−^* (D) and *Ulnaless* (B, E) forelimbs were hybridized with *Runx2* antisense riboprobe. In control forelimbs, *Runx2* is expressed in the condensing cartilage anlagen of the zeugopod at E12.5 (A) and in early hypertrophic chondrocytes (C, yellow arrow) and the perichondrium at E14.5 (C, red arrow). In the ulna of both E14.5 *Hox* mutants, *Runx2* expression cannot be detected in chondrocytes, but in the perichondrium (D, E, red arrows). In E12.5 *Ulnaless* mice, *Runx2* expression is restricted to the region of the future, posterior perichondrium (B, red arrow). 80x magnification; R = radius, U = ulna.

**Figure 6:**
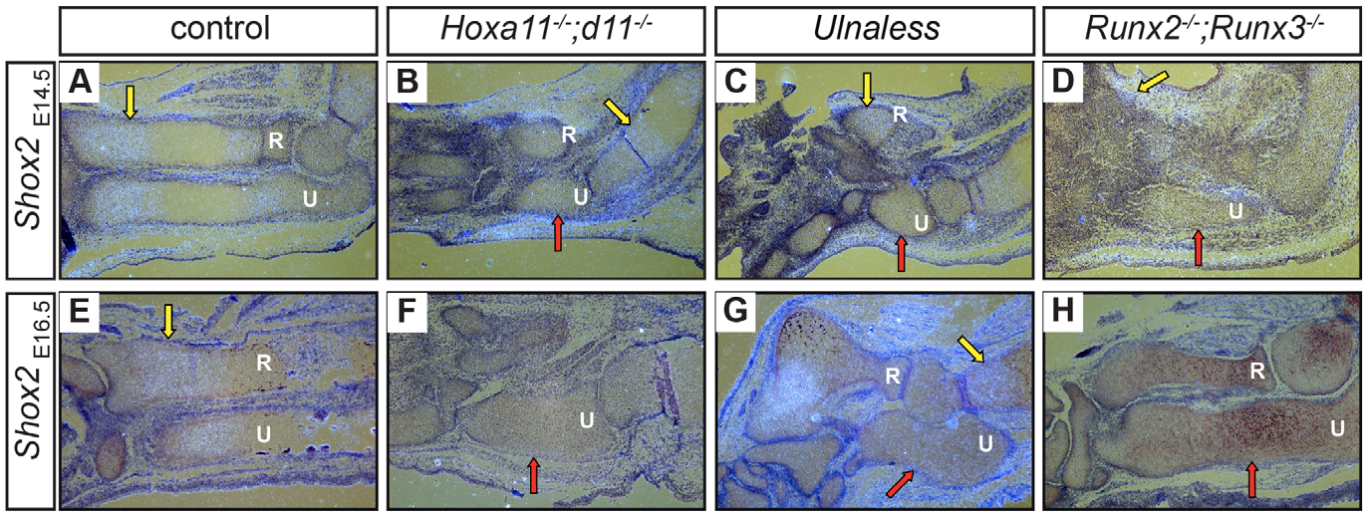
Shox2 acts downstream of both Hoxa11 and Hoxd11 as well as of Runx2. *Shox2 in situ* hybridization on E14.5 (A–D) and E16.5 (E–H) control (A, E), *Hoxa11^−/−^;d11^−/−^* (B, F), *Ulnaless* (C, G) and *Runx2^−/−^;Runx3^−/−^* (D, H) forelimbs revealed the absence of its expression in the ulna of both *Hox* mutants (B, C, F, G, red arrows) and in ulna and radius of *Runx2^−/−^;Runx3^−/−^* mutant mice (D, H, red arrows). However, *Shox2* expression was detected in other regions of the analyzed forelimbs (B, C, D, G, yellow arrows). 80x magnification (A–C, E–H), 100x magnification (D); R = radius, U = ulna.

Mice deficient for *Runt related transcription factor 2* (*Runx2*) and *Runx3* and mice carrying a limb specific deletion of the gene *Short stature homeobox 2* (*Shox2*) exhibit a similar phenotype as *Hoxa11^−/−^;d11^−/−^* and *Ulnaless* mice. Both show shortened limbs, an arrest in chondrocyte differentiation and the absence or severe reduction of *Ihh* expression [37], [38]. Runx2 and Shox2 are thus good candidates to mediate the effect of *Hox* genes in chondrocyte differentiation. To test this hypothesis, we analyzed the expression of *Runx2* and *Shox2* in the ulna of the E14.5 *Hox* mutants by *in situ* hybridization. In E14.5 wild type limbs, *Runx2* is expressed in early hypertrophic chondrocytes (Fig. 5C, yellow arrow) and in the perichondrium [39] (Fig. 5C, red arrow), whereas *Shox2* is expressed in proliferating chondrocytes at E14.5 and E16.5 [40] (Fig. 6A, E, yellow arrows). In contrast, neither *Runx2* nor *Shox2* expression can be detected in chondrocytes of the ulna of either mutant (Fig. 5D, E; Fig. 6B, C, F, G, red arrows), although both genes are strongly expressed in other cartilage anlagen of the forelimbs (Fig. 5D, E, yellow arrows; Fig. 6B, C, G, yellow arrows).

Interestingly, in addition to being expressed in hypertrophic chondrocytes, *Runx2* has been described to be expressed in the cartilage condensations [41], [42]. To investigate if this early expression is regulated by *Hox* genes, we analyzed the expression of *Runx2* at E12.5. In wild type limbs, strong *Runx2* expression was detected in the condensing cartilage anlagen of the forelimb zeugopod (Fig. 5A). In contrast, already at this early stage, *Runx2* expression is severely reduced in the condensing chondrocytes of the ulna of *Ulnaless* mutants and could only be detected in the region of the future perichondrium (Fig. 5B, red arrow). This restriction of the *Runx2* expression to the perichondrium was observed in the E14.5 ulna of both *Hox* mutants as well (Fig. 5D, E, red arrows).

To further investigate the epistatic relationship of Runx2 and Shox2, we analyzed the expression of *Shox2* in E14.5 *Runx2* mutants. As Runx2 and Runx3 have highly redundant functions during chondrocyte differentiation and compound mutants display a similar arrest in chondrocyte differentiation as *Hoxa11^−/−^;d11^−/−^* and *Ulnaless* mutants [37], we used *Runx2^−/−^;Runx3^−/−^* double mutant forelimbs for this analysis. Although we detected strong expression of *Shox2* in the surrounding limb tissue (Fig. 6D, yellow arrow), its expression was severely reduced in chondrocytes at E14.5 (Fig. 6D, red arrow). To exclude that the lack of *Shox2* expression reflects a delay in development, we analyzed *Runx2^−/−^;Runx3^−/−^* as well as *Hoxa11^−/−^;d11^−/−^* and *Ulnaless* forelimbs at E16.5, but could not detect expression of *Shox2* in the ulna of either mutant (Fig. 6F–H, red arrows). It is thus likely that early *Runx2/Runx3* expression acts upstream of Shox2.

The role of Runx2 in inducing hypertrophic differentiation has been intensively studied [39]. The loss of *Ihh*-expressing cells in the *Hox* mutants is likely due to the absence of this inducer of hypertrophic differentiation. Furthermore, previous experiments indicated that *Shox2^c/−^* mutants show reduced *Runx2* expression in the humerus [38], [40] placing Shox2 upstream of *Runx2*. Interestingly, our investigation of *Runx2^−/−^;Runx3^−/−^* double mutants revealed a lack of *Shox2* expression placing Runx2 upstream of *Shox2*. This is supported by our expression analysis, in which we detect *Shox2* expression in chondrocytes starting from E13.5 (data not shown), whereas the early expression of *Runx2* can already be detected at E12.5 (Fig. 5A).

Runx2/Runx3 and Shox2 seem thus to interact in a complex relationship downstream of *Hox* genes in chondrocytes. At early stages Runx2/Runx3 seem to act upstream of *Shox2*, whereas at later stages Shox2 appears to act upstream of *Runx* genes. If Runx2/Runx3 induce the differentiation of round and columnar cells, which in turn express *Shox2*, or if Shox2 together with Runx2/Runx3 is required to drive the chondrocyte differentiation program has to be investigated in future experiments. In this respect it is interesting to note that loss of *Runx2* affects hypertrophic differentiation strongest in the humerus [39], but loss of *Runx2* and *Runx3* inhibits differentiation in all skeletal elements of the limbs [37]. Similarly, the defects in *Shox2^c/−^* mice are strongest in humerus and femur [38]. Interestingly, humans express the two paralog genes, SHOX and SHOX2, whereas mice only contain Shox2. Mutations in SHOX are associated with Léri-Weill dyschondrosteosis [43], Turner syndrome [44] and Langer mesomelic dysplasia [45], which are associated with pronounced limb defects in the zeugopod. Which gene replaces the role of SHOX in mice and if deletion of both would affect zeugopod and stylopod to similar degrees remains a question for future studies.

### Conclusions

**Figure 7:**
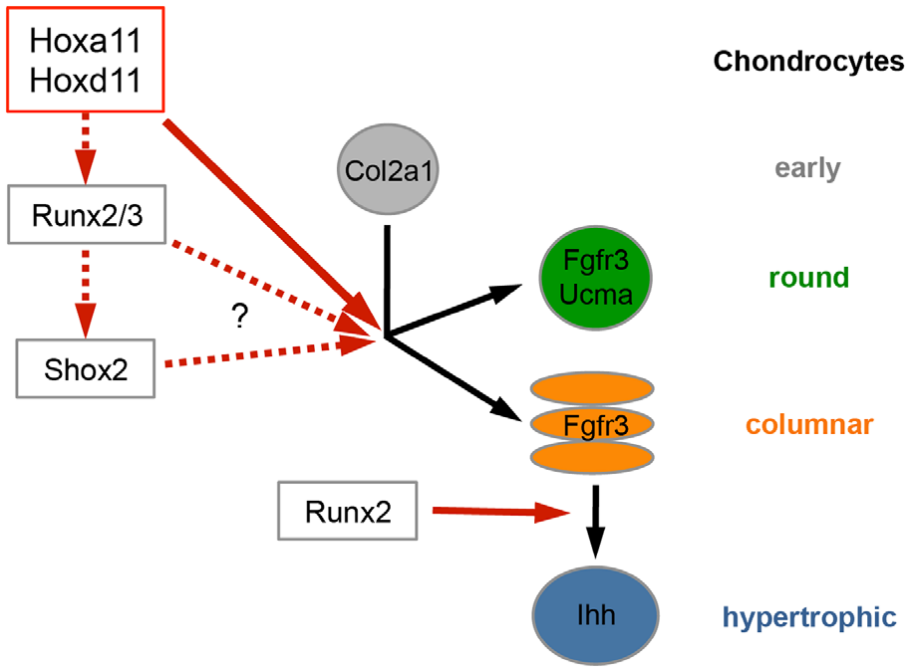
Model for the regulation of chondrocyte differentiation by posterior *Hox* genes. Hoxa11 and Hoxd11 regulate the differentiation into round and columnar chondrocytes upstream of the transcription factors Runx2 and Shox2. Black arrows: chondrocyte differentiation steps; red arrows: gene regulation, dashed arrows: predicted epistatic relationship.

Our analysis demonstrates that Hoxa11 and Hoxd11 regulate an early step of chondrocyte differentiation in the zeugopod of mice (Fig. 7). Although the differentiation of mesenchymal cells into *Col2a1*-expressing chondrocytes is not significantly affected by loss of Hoxa11 and Hoxd11, chondrocytes fail to undergo differentiation into *Ihh-*expressing hypertrophic chondrocytes. Here we further define the stage at which chondrocyte differentiation is arrested. We show that, although the condensation of the skeletal elements is not obviously affected at E12.5, chondrocytes stay arrested at an early step in the differentiation program, before the differentiation into columnar and round chondrocytes takes place. In addition, our data identify a new chondrocyte subtype expressing low levels of *Fgfr3* but no *Ucma*, which is not competent to undergo hypertrophic differentiation in absence of Ihh signaling.

We further demonstrate that *Hox* genes act upstream of the transcription factors *Runx2* and *Shox2,* two key regulators of the chondrocyte differentiation program (Fig. 7), which are expressed in most cartilage anlagen of wild type long bones. The observation that their expression is specifically affected in the ulna of the Hox mutants make them likely candidates to translate the positional information established by the ulna-specific “*Hox* code” into a skeletal pattern. As individual skeletal elements seem to react with different sensitivity to changes in the expression of *Runx2, Runx3* and *Shox2*, fine-tuning their expression by specific combinations of *Hox* genes might provide a mechanism to modulate size and shape of a cartilage element.

## Materials and Methods

### Ethics Statement

Mice were kept and bred according to the institutional guidelines of the University Duisburg-Essen and the University Hospital Essen, specifically approved by the animal welfare officer of the University Duisburg-Essen. Mouse husbandry was approved by the city of Essen (Az: 32-2-11-80-71/203) in accordance with § 11 (1) 1a of the “Tierschutzgesetz”. Work with transgenic animals was approved by the “Bezirksregierung Duesseldorf” (Az: 53.02.01-D-1.57/09, Anlagen-Nr. 965) in accordance with § 8 Abs. 4 Satz 2 GenTG of the “Gentechnikgesetz”.

### Mice and Genotyping

For time pregnancies, noon of the day, when a vaginal plug was observed, was considered to be embryonic day (E) 0.5. Double mutant *Hoxa11^−/−^;d11^−/−^* embryos were generated by mating compound heterozygous parental mice as described before [20], [21]. Embryos with at least one wild type allele for each gene were used as a control. Genotyping was performed by three primer-PCR with the following primers: *Hoxa11*for: 5′-gctggcttttatctgaagccgg-3′, *Hoxa11*rev: 5′-ctcccaattccagtaggctgg-3′, *Hoxa11*Neo: 5′-ggttgttcagactacaatctgacc-3′, *Hoxd11*for: 5′-cctttttcctatctcagtgccag-3′, *Hoxd11*rev: 5′-ggggtacatcctggagttctca-3′, *Hoxd11*Neo: 5′-ttcaagcccaagctttcgcgag-3′. *Ulnaless* mice were obtained from The Jackson Laboratory (Bar Harbor, Maine) and kept in the B6EiC3 background. Heterozygous *Ulnaless* embryos were generated by mating heterozygous *Ulnaless* females with wild type males of the *Ulnaless* colony. Wild type embryos of *Ulnaless* matings were used as controls. Genotyping of the *Ulnaless* allele was performed by PCR with the following primers: *Ul-*for: 5′-acccttggactaaagaccaaa-3′, *Ul-*rev: 5′-ttgctgtaaactcatcaggaag-3′. *Runx2^−/−^;Runx3^−/−^* mice have previously been described [37]. *Runx2^+/−^;Runx3^+/+^* and *Runx2^+/−^;Runx3^+/−^* embryos were used as a control. Genotyping for *Runx2* was performed by using the following primers: *Runx2-*wt-for: 5′-cttgaaggccacgggcag-3′, *Runx2-*wt-rev: 5′-agcgacgtgagcccggtg-3′, *Runx2-*Neo-for: 5′-tctggattcatcgactgtgg-3′, *Runx2-*Neo-rev: 5′-cttgaaggccacgggcag-3′. *Runx3* genotyping was performed as previously described [37].

### Histological Analysis and *in situ* Hybridization

Embryos were dissected in phosphate buffered saline (PBS). Forelimbs were fixed overnight in 4% paraformaldehyde (PFA) at 4°C, dehydrated and embedded in paraffin wax. Serial sections of 5 µm were stained or used for *in situ* hybridization. Safranin-Weigert staining was performed with a series of Weigert’s hematoxylin (Roth), 0.1% fast green (Sigma) and 0.1% safranin O (Sigma) according to the staining procedure [46]. For radioactive *in situ* hybridization, antisense riboprobes were labeled with [P^33^]-UTP (Hartman Analytic). Hybridization was performed in 50% formamide at 70°C as described previously [30]. Developed slides were counterstained with 0.2% toluidine blue O (Sigma) in 1% sodium borate. The following probes were used for *in situ* hybridization: *Ihh* [47], *Ptch1* [48], *Fgfr3* [35], *Ucma* [36], *Col2a1* [49], *Col10a1* [50], *Runx2* [42]. The *Shox2* probe was amplified from cDNA of E15.5 wild type limbs using primers *Shox2*-for: 5′-ttgcaacgtgacgcccttgtc-3′, *Shox2-*rev: 5′-ggcgctatccacttctcactg-3′ and cloned into pCR4-TOPO (Invitrogen).

Unless otherwise indicated, forelimbs of at least two individual mice were analyzed by Safranin-Weigert staining or hybridized with the same antisense probe showing the same result.

### Limb Explant Cultures

Forelimbs of E13.5 *Ulnaless* embryos were dissected by removing skin and muscle. The limbs were cultured for 4 days in Biggers or BGJb medium (Invitrogen) supplemented with 1% antibiotic/antimycotic (Invitrogen), 0.1% bovine serum albumin (BSA) (Roth) and 1.4 mM glutamine (Invitrogen) in *in vitro* fertilization dishes (BD Biosciences Falcon) at 37°C, 5% CO_2_. One forelimb of the embryo was treated with 10 µM cyclopamine (Calbiochem), whereas the other forelimb was used as untreated control. The medium was changed every day. The limbs were fixed for 3 hours in 4% PFA at 4°C and embedded in paraffin wax.

### Imaging

Bright- and dark field pictures were taken with the camera SPOT 14.2 using the SPOT advanced software, version 4.5.7 (Diagnostic Instruments). *In situ* hybridization signals were visualized in either white or red using dark field microscopy with the illuminator Intralux 5000-1 (Volpi).

## Supporting Information

Figure S1 **Chondrocyte differentiation is initiated normally in the zeugopod of** ***Ulnaless*** **mice.** *In situ* hybridization on E12.5 control (A) and *Ulnaless* (B) forelimbs with a *Col2a1* antisense riboprobe reveals no difference in the *Col2a1* expression in *Ulnaless* forelimbs compared to control (A, B). 80x magnification; R = radius, U = ulna.
(TIF)
