# Supplementary figures and images for: Hoxa11 and Hoxd11 Regulate Chondrocyte Differentiation Upstream of Runx2 and Shox2 in Mice
Source: PLoS One. 2012 Aug 20;7(8):e43553. doi: 10.1371/journal.pone.0043553 (PMC3423364; doi:10.1371/journal.pone.0043553)

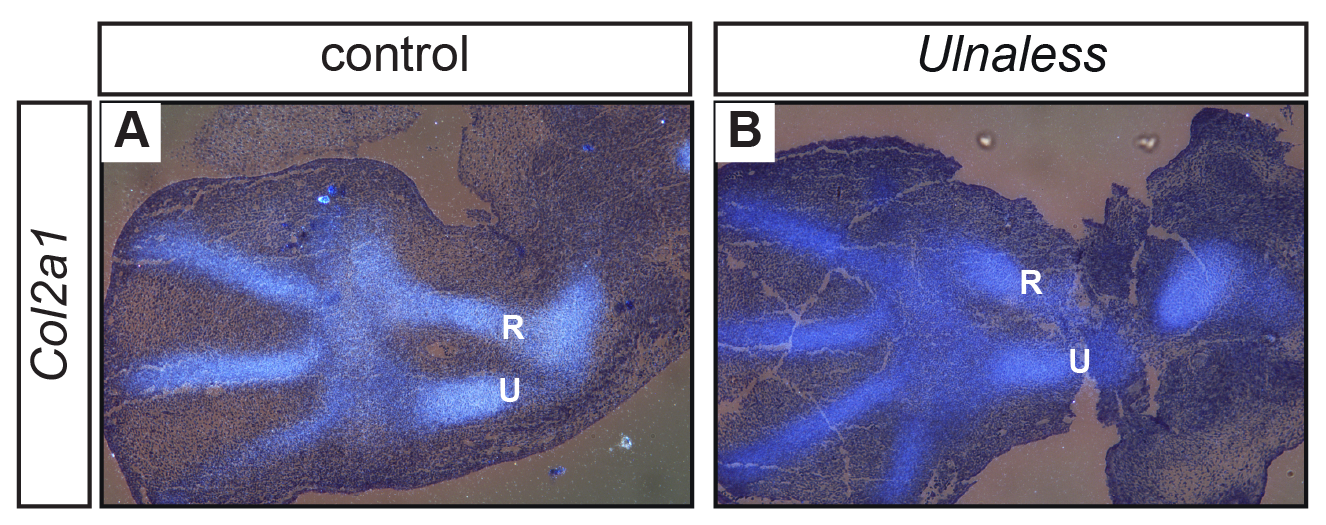

Supplement: Figure S1 — Chondrocyte differentiation is initiated normally in the zeugopod of Ulnaless mice. In situ hybridization on E12.5 control (A) and Ulnaless (B) forelimbs with a Col2a1 antisense riboprobe reveals no difference in the Col2a1 expression in Ulnaless forelimbs compared to control (A, B). 80x magnification; R = radius, U = ulna. (TIF) [file pone.0043553.s001.tif]
